# Covid-19 Outbreak In Italy: Are We Ready for the Psychosocial and the Economic Crisis? Baseline Findings From the PsyCovid Study

**DOI:** 10.3389/fpsyt.2020.00556

**Published:** 2020-06-10

**Authors:** Chiara Cerami, Gaia C. Santi, Caterina Galandra, Alessandra Dodich, Stefano F. Cappa, Tomaso Vecchi, Chiara Crespi

**Affiliations:** ^1^ Center for Neurocognition, Epistemology and Theoretical Syntax (NETS), Scuola Universitaria Superiore IUSS Pavia, Pavia, Italy; ^2^ IRCCS Mondino Foundation, Pavia, Italy; ^3^ Istituti Clinici Scientifici Maugeri IRCCS, Pavia, Italy; ^4^ Center for Neurocognitive Rehabilitation—CIMeC, University of Trento, Rovereto, Italy; ^5^ Department of Brain and Behavioral Sciences, University of Pavia, Pavia, Italy

**Keywords:** Covid-19 outbreak, perceived impact on health, economic crisis, psychosocial frailty, loneliness, empathy, distress, coping abilities

## Abstract

The Covid-19 pandemic is burning all over the world. National healthcare systems are facing the contagion with incredible strength, but concern regarding the psychosocial and economic effects is growing quickly. The PsyCovid Study assessed the influence of psychosocial variables on individual differences from the perceived impact of the Covid-19 outbreak on the issues of health and economy in the Italian population. Italian volunteers from different regions completed an online anonymous survey. The main outcomes were the perceived impact of the Covid-19 outbreak on health and the economy. A two-way MANOVA evaluated differences in the main outcomes, with geographical area (northern, central, and southern regions) and professional status (healthcare workers or not) as factors. We then tested the relationship linking psychosocial variables (i.e. perceived distress and social isolation, empathy, and coping style) to the main outcomes through two different mediation models. 1163 responders completed the survey (835 females; mean age: 42 ± 13.5 y.o.; age range: 18-81 y.o.) between March 14 and 21, 2020. Healthcare workers and people living in northern Italy reported a significantly worse outbreak impact on health, but not on the economy. In the whole sample, distress and loneliness were key variables influencing the perceived impact of the Covid-19 outbreak on health, while empathy and coping style affected the perceived impact on the economy. The Covid-19 pandemic is a worldwide emergency in terms of psychological, social, and economic consequences. Our data suggests that in the Italian population, actual differences in individual perception of the Covid-19 outbreak severity for health are dramatically modulated by psychosocial frailty (i.e., distress and loneliness). At the same time, problem-oriented coping strategies and enhanced empathic abilities increase people's awareness of the severity of the impact of the Covid-19 emergency on economics. There is an immediate need for consensus guidelines and healthcare policies to support interventions aimed to manage psychosocial distress and increase population resilience towards the imminent crisis.

## Introduction

**Figure 1:**
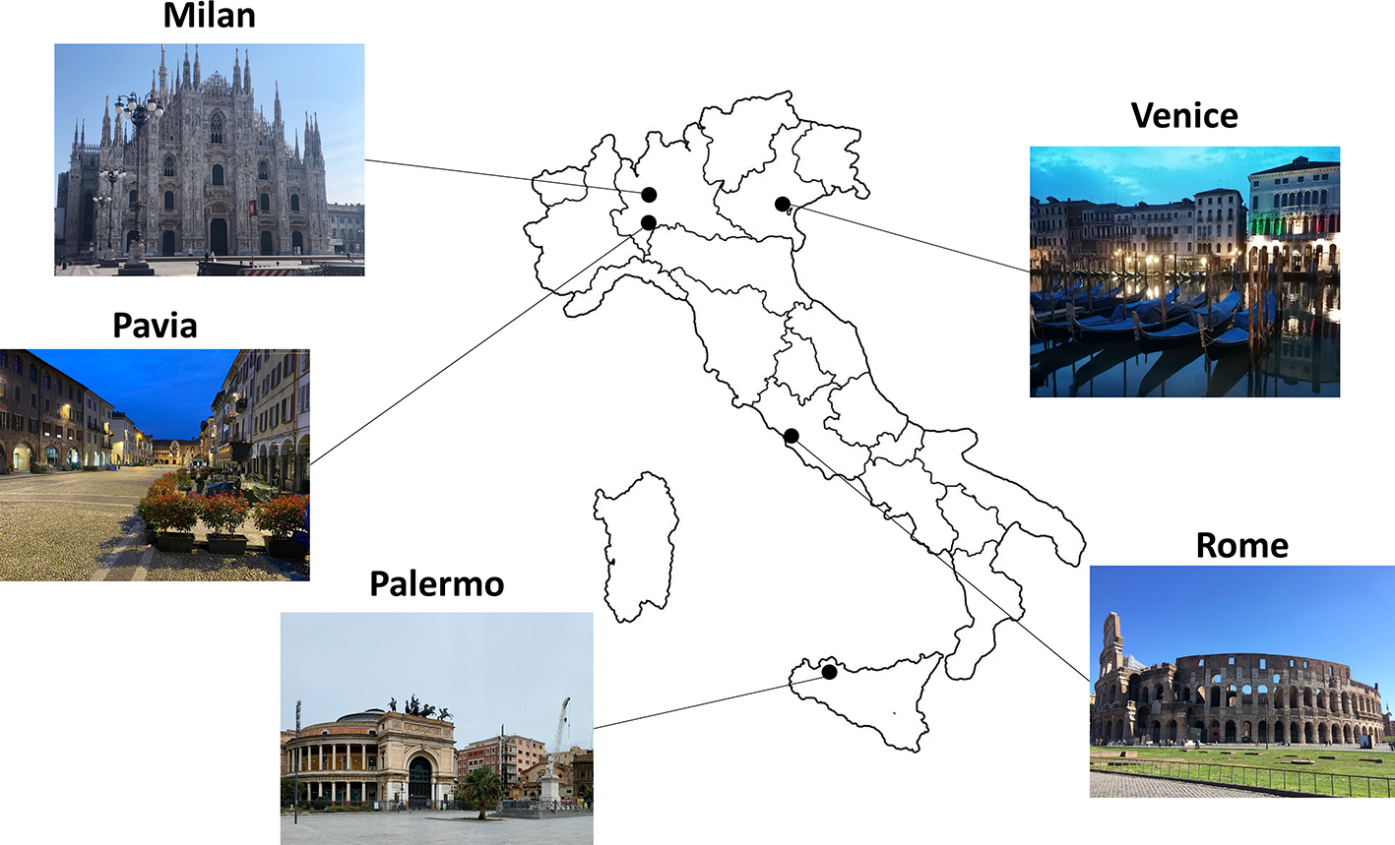
Italian ghost towns. The figure illustrates the effect of social distance measures in the Italian cities of Milan, Pavia, Venice, Rome, and Palermo.

In late 2019, pneumonia cases of an unknown etiology, then proven to be caused by a new coronavirus (2019-nCOV or SARS-CoV2), appeared in Wuhan, Hubei Province of China. The first clusters of patients were epidemiologically linked to human-animal transmission in the Huanan Seafood Wholesale Market of Wuhan. At the end of December 2019, the World Health Organization (WHO) was alerted of the novel viral illness that caused respiratory symptomatology which sometimes resulted in severe acute respiratory syndrome (SARS) (1, 2). The first two cases in Italy, a couple of Chinese tourists, were registered in Rome (3) at the end of January 2020. Later they were proved to be infected prior to their arrival in Italy (4). Specific algorithms and protocols were then applied, and specialized teams were instituted, in order to control the contagion spread (5). However, a dramatic increase of positive cases and hospitalizations quickly followed, especially in Northern Italy. After the confirmation of 2019-nCov positivity of the two Chinese tourists admitted at the Spallanzani Hospital in Rome on February 21, 2020, the Italian government declared a state of emergency (6). Extraordinary measures to prevent the virus spread were instituted only on March 9, 2020 (DCPM #iorestoacasa – I stay at home), and further hardened on March 13, 20, and 22, 2020. At that time, social distancing became extreme and unprecedented (**Figure 1**). A daily press release system was established and educational campaigns were launched, in order to sensitize and encourage people to undertake contact precautions and avoid the contagion. The Covid-19 outbreak disruptively changed habits, routines, and lifestyles, affecting human relationships and the productivity of the entire country. Roads and streets were deserted and the suspicion of infection from others is high. At the time of the last revision of this manuscript (May 13, 2020) in Italy there were 222.104 confirmed case and 31.106 deaths.

A few days after March 9, 2020, we started the PsyCOVID longitudinal study. We designed this psychosocial research study, taking into account three key requirements to test the impact of infectious diseases (7): i) a systemic perspective, directed to the general public, designed to be as inclusive as possible; ii) a prospective outlook, including a baseline assessment during the social restrictive measures and two follow-ups (the first a month after the abolition of these measures, and the second six months after the first follow-up); iii) measurable outcomes of psychosocial variables, suitable to detect fragile sub-populations who would benefit from specific interventions at the end of the outbreak.

Indeed, extreme social restrictions like social distancing, as well as emergency situations and settings that healthcare professionals have to face every day, require individuals to allocate enormous resources to the process of psychosocial adjustment to such a novel and catastrophic situation, which in the long-term may exert a critical impact on individuals' well-being, mental health, and quality of life. In such a context, increased distress and loneliness, possibly emerging as a result of social isolation, can profoundly affect our perception of events and, importantly, may exacerbate the risk of negative mental health outcomes, including the emergence or the worsening of anxious and depressive symptoms, addictive behaviors, thought disorders, as well as the increase of the risk of suicide (8).

At the same time, effective coping strategies and empathic abilities can help individuals to enhance their awareness of the problem, build resilience, and increase social responsibility, and thus face such a complex situation in a more constructive way.

In this paper, we report findings on the baseline assessment of the PsyCOVID study aiming at evaluating differences in the perceived impact of the Covid-19 outbreak on health and economy in the Italian population during the very first days of the extreme social distancing measures, specifically taking into account the impact of demographic variables, regional differences (Northern, Central, and Southern regions), and professional status (healthcare workers or not).

## Participants and Methods

### Participants

Between Mar 14 and 21, 2020, we conducted an anonymous online survey among adult Italian residents. Study protocol was approved by the University Ethics Committee (IUSS—University of Pavia). We selected convenience sampling, selecting participants based on their accessibility and proximity to the research group. We created the survey using Google Forms and distributed it through a link, accessible to anyone (https://forms.gle/5f3yH3aTNJYEuJ7B9). We distributed the survey link *via* written invitations through e-mails, Whatsapp, and social network messaging (Facebook, Instagram, and Linkedin). Then, we asked initial participants to diffuse the questionnaire through their social networks. The eligibility criteria were age (18 years of age or older), ability to provide an informed consent, and place of residence (Italy). At the beginning of the survey, we presented the study objective and timeline, the commitment required of participants, and information about the research team. We asked potential participants to read and provide their informed consent by clicking a box. After providing informed consent, participants were directed to the survey. We first invited all participants to provide a reference in order to be contacted for the following phases. Participants did not receive any incentive to take part in the study. The response rate was 98%. We calculated the rate response as the ratio of the number of complete responders to the total number of potential participants who had the chance to access the first page of the study. Non-responders were persons who did not provide their informed consent to participate or who declared an age < 18 years old.

A total of 1,163 adult Italian residents completed the survey (72% females; mean age: 42 ± 13.5 y.o.; age range: 18–81 y.o.). The majority (65.6%) of participants were residents in Northern Italy, 9.6% in Central Italy, and 24.8% in Southern Italy. Of all responders, 14.3% were healthcare professionals. **Table 1** provides details about the socio-demographic characteristics of the sample.

**Table 1:** Demographic information.

Characteristics	No. (and %) of respondents
**Sex**	
Male	326 (28.0)
Female	837 (72.0)
**Age**	
Youth age (18-24 y)	61 (5.2)
Young adults (25-39 y)	528 (45.4)
Adults (40-64 y)	475 (40.9)
Elderly (>65 y)	99 (8.5)
**Education**	
Secondary school (8 y)	26 (2.2)
High school (13 y)	323 (27.8)
Graduate school (16-18 y)	549 (47.2)
Postgraduate school (>18 y)	265 (22.8)
**Occupation**	
Student	84 (7.2)
Housewife	31 (2.7)
Unemployed	48 (4.1)
Employee	558 (47.9)
Manager	96 (8.3)
Freelance	211 (18.1)
Professor or Researcher	32 (2.8)
Retired	103 (8.9)
**Job field**	
Industry	106 (9.1)
Financial and Economy	109 (9.4)
Communication Industry	57 (4.9)
Art and Manufacturing	55 (4.7)
Humanistic	188 (16.2)
Non-profit	90 (7.7)
Construction	22 (1.9)
Trade	58 (5.0)
Healthcare	165 (14.3)
Education and University	56 (4.8)
Public Services	54 (4.6)
Others	203 (17.4)
**Geographic Area (place of birth)**	
Norther Italy	646 (55.5)
Centre Italy	111 (9.5)
Southern Italy	375 (32.3)
Abroad	31 (2.7)
**Geographic Area (place of residence)**	
Norther Italy	763 (65.6)
Centre Italy	112 (9.6)
Southern Italy	288 (24.8)
**Size of place of residence**	
Rural area (<1k people)	11 (0.9)
Small-size town (1-10k people)	202 (17.4)
Medium-size town (10-50k people)	314 (26.9)
Small-size city (50-250k people)	243 (20.9)
Medium-size city (250-500k people)	46 (4.0)
Big-size city (500k-1mln people)	142 (12.2)
Metropolis (>1 mln people)	205 (17.7)

The table reports demographic features of the PsyCOVID study baseline sample (N=1,163) collected within the first week after the start of the study (March 14–21, 2020).

### Measures

The questionnaire collected data on socio-demographic characteristics (**Table 1**), an assessment about the perceived impact of the Covid-19 outbreak on health and the economy (main outcome measures), and psychosocial factors.

#### Outcome Measures: Assessment of the Perceived Impact of Covid-19 Outbreak

We assessed the perceived impact of the Covid-19 outbreak with 4 items for health (*average interitem covariance*=0.34; *Cronbach's alpha or α = 0*.*74*) and 4 items for the economy (*average interitem covariance*=0.31; *α = 0*.*81*). Items on both the health and economy scales required participants to rate the perceived severity of the Covid-19 outbreak at the local (item 1: city or town), regional (item 2), and global (item 3: national; item 4: international) levels, on a 5-point Likert scale (0=not serious at all; 4=extremely serious). Finally, for each scale we created an index (range 0-16), obtained by summing up the item ratings within each scale. We used the resulting measures as outcome variables in our subsequent analyses.

#### Psychosocial Predictors

In the PsyCOVID study we decided to evaluate a set of specific psychosocial dimensions related to emergency settings and situations, including perceived global distress (9, 10), loneliness (10), empathic skills (11, 12), and coping strategies (13). To collect information about these psychosocial dimensions we used a battery of validated questionnaires in the Italian language. In particular, we assessed the different facets of global distress with the Italian version of the Depression Anxiety Stress Scales-21 (14), allowing participants to obtain specific sub-scores of depression, anxiety, and stress. We used the Empathic Concern and Perspective Taking sub-scales of the Interpersonal Reactivity Index [IRI (15)] to capture emotional and cognitive facets of empathic abilities. Loneliness was assessed with the Italian Loneliness Scale (16), including the three sub-scales (Emotional, Social, and General Loneliness). Finally, coping strategies were investigated with the short version of the Italian version of the Coping Orientation to the Problems Experienced [COPE-NVI-25 (17)], measuring different coping behaviors or styles towards problems and stressful events, reflected in 5 scale sub-scores (Positive attitude, Problem orientation, Transcendence orientation, Social support, Avoidance strategies).

### Statistical Analysis

Since fewer than 2% of cases were missing in any analysis, we dropped cases with missing values *via* list-wise deletion. We set statistical significance at p <0.05 for all statistical tests we performed. We calculated descriptive statistics including frequencies and percentages for categorical variables, and mean and standard deviation for pseudo-continuous variables. We estimated group differences in the perceived impact of the Covid-19 outbreak on health and the economy dimensions with a two-way MANOVA, considering geographical area (northern, central, and southern regions) and professional status (healthcare professionals vs. non-healthcare professionals) as factors. We additionally described between-group differences on psychosocial variables, with geographical area (one-way ANOVA) and professional status (Student's t-test) as grouping variables in separate analyses. We then explored the correlations (Pearson's r coefficient) between psychosocial variables and main outcomes.

Finally, based on correlation results, we tested two mediation models. The first (*Model 1*) tested the indirect effect of perceived distress (Stress subscale of the DASS-21) on the relationship between loneliness (General Loneliness subscale of the ILS) and the perceived impact of Covid-19 outbreak on health. The second mediation model (*Model 2*) assessed the indirect effect of coping style (Problem orientation sub-score) on the relationship between empathic skills (Composite score of Empathic Concern and Perspective taking sub-scales of the IRI) and the perceived impact of the Covid-19 outbreak on the economy.

We carried out sample description and statistical group analyses using SPSS (https://www.spss.it/), and tested mediation models using STATA (https://www.stata.com/).

## Results

**Figure 2:**
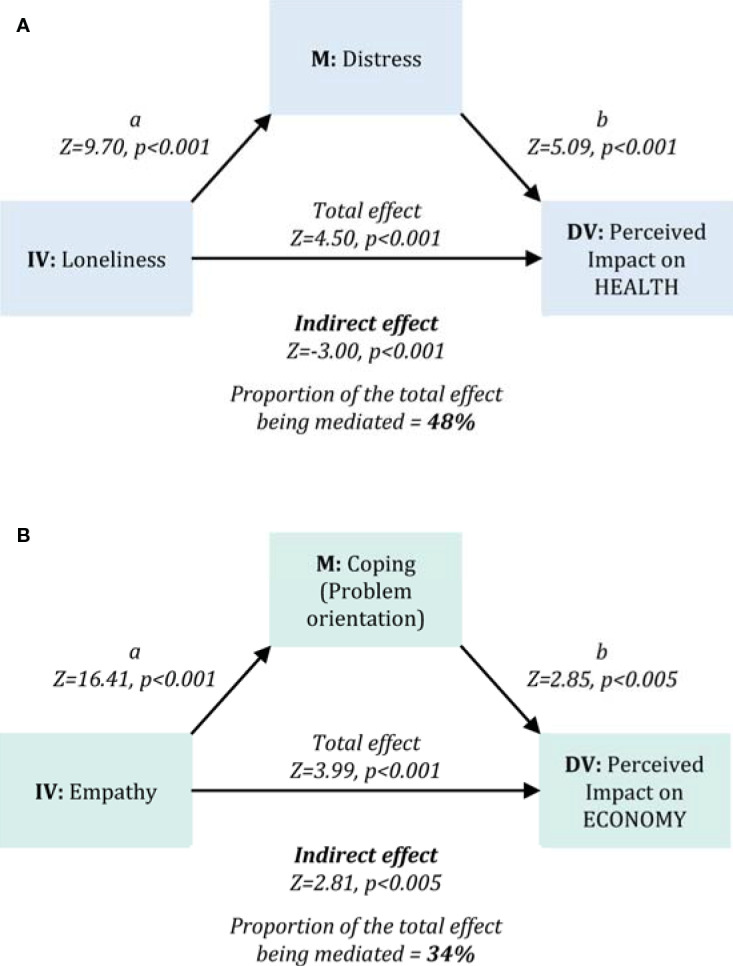
Mediation analyses. The figure illustrates the two mediation models tested for the main outcomes related to the perceived impact of the COVID-19 outbreak for Health (*Model 1*, Panel **A**) and for Economy (*Model 2*, Panel **B**). *Model 1* assessed the mediation effect of perceived distress (DASS-21 Stress sub-scale) on the relationship between perceived loneliness (ILS General Loneliness sub-scale) and the perceived impact of the COVID-19 outbreak on health. *Model 2* assessed the mediation effect of problem-oriented coping strategies (COPE-NVI-25 Problem orientation sub-scale) on the relationship between empathy (IRI Empathic concern and Perspective Taking sub-scales) and the perceived impact of the COVID-19 outbreak on the economy. *Figure acronyms: IV, Independent variable; DV, Dependent variable; M, mediator*.

Descriptive statistics are illustrated in **Tables 1** and **2** and in **Figure 2**.

**Table 2:** Group comparisons on psychosocial variables.

	A. Professional status		B. Geographic area		
	*Healthcare*	*Non-healthcare*	*Northern Italy*	*Central Italy*	*Southern Italy*
*DASS-21 Depression*	3,55 ± 3,65	3,52 ± 3,87	3,44 ± 3,65	3,75 ± 4,33	3,64 ± 4,11
*DASS-21 Anxiety*	1,84 ± 2,46	2,07 ± 2,87	1,94 ± 2,64	2,01 ± 3,20	2,27 ± 3,09
*DASS-21 Stress*	6,21 ± 4,23	5,32 ± 4,31	5,60 ± 4,15	5,21 ± 4,66	5,09 ± 4,55
*ILS Emotional*	7,66 ± 4,38	7,61 ± 4,35	7,54 ± 4,31	8,12 ± 4,42	7,64 ± 4,44
*ILS Social*	13,55 ± 4,22	13,44 ± 4,39	13,38 ± 4,40	13,67 ± 4,08	13,59 ± 4,37
*ILS General*	8,48 ± 5,11	8,40 ± 5,07	8,31 ± 5,07	8,66 ± 5,04	8,63 ± 5,15
*IRI Empathic Concern*	20,57 ± 3,82	20,39 ± 4,10	20,35 ± 4,09	20,63 ± 3,94	20,47 ± 4,06
*IRI Perspective Taking*	18,80 ± 4,31	18,23 ± 4,57	18,12 ± 4,55	18,54 ± 4,33	18,72 ± 4,56
*COPE-NVI-25 Positive attitude*	24,32 ± 5,38	23,74 ± 5,48	23,79 ± 5,31	23,13 ± 5,25	24,16 ± 5,97
*COPE-NVI-25 Social support*	20,51 ± 5,03	19,10 ± 5,33	19,44 ± 5,16	18,74 ± 5,55	19,09 ± 5,59
*COPE-NVI-25 Problem orientation*	21,72 ± 4,42	20,66 ± 4,75	20,83 ± 4,53	19,96 ± 4,20	21,05 ± 5,35
*COPE-NVI-25 Transcendence orientation^§^*	8,82 ± 5,95	9,39 ± 6,33	8,56 ± 5,93	8,87 ± 5,82	11,42 ± 6,87
*COPE-NVI-25 Avoidance strategies*	9,40 ± 3,65	10,25 ± 3,85	9,96 ± 3,61	10,48 ± 3,93	10,44 ± 4,31

The table illustrates group comparisons on psychosocial variables assessed, taking into account professional status **(A)** and geographic area **(B)**. For each group we report mean and standard deviation. Statistical significance at p < 0.05 is indicated with (*) for group comparisons based on professional status and with (§) for group comparisons based on geographic area.

The two-way MANOVA showed a significant multivariate effect of both geographic area (Λ=0.955 F(4, 2308)=13.582, p<0.001) and professional status (Λ=0.991 F(2, 1154)=5.042, p=0.007) on the perceived severity of the Covid-19 outbreak. However, the interaction between geographic area and professional status was not significant (Λ=0.996; F(4, 2308)=1.031, p=0.390). Univariate results revealed that both geographic area and professional status had a significant effect on the perceived severity for health (*geographic area:* F(2, 1161)=19.391, p<0.001; *professional status*: F(2,1161)=30.920, p=0.035), but not for the economy (*geographic area:* F(2, 1161)=0.231, p=0.794; *professional status*: F(2,1161)=0.874, p=0.350).

*Post hoc* tests (Tukey HSD) on geographical area showed the perceived severity for health in northern Italy was significantly different from that of central (p<0.001) and southern regions (p<0.001). The perceived outbreak impact on health was significantly higher (i.e., more serious) in healthcare workers and in people living in northern Italy, compared to non-healthcare workers and people living in central-southern Italian regions.

Group comparisons on all psychosocial variables by professional status did not show significant results (**Table 2**). The same was true for group comparisons based on geographical area, with the exception of coping strategies reflecting transcendence orientation, which characterize southern regions more than central (Tukey HSD, p=0.001) and northern ones (Tukey HSD, p<0.001).

Correlation analyses assessing the relationship between main outcomes and psychosocial variables are reported in **Table 3**. Several variables were significantly related to one or both study outcomes. On this basis, we selected a small set of variables to test two mediation models. In *Model 1* (**Figure 2A**), we tested the mediation effect of perceived distress – positively correlated to the dependent variable and negatively with the independent variable – on the positive relationship linking general loneliness (independent variable) and the perceived impact of the Covid-19 outbreak for health (dependent variable) (direct effect: Z=-4.32, p<0.001). Results highlighted a significant indirect effect of perceived distress (Z=4.50 p<0.001), mediating approximately 48% of the total effect of loneliness on the perceived impact of the Covid-19 outbreak for health. In *Model* 2 (**Figure 2B**), we tested the mediation effect of problem-oriented coping strategies – positively correlated with both the dependent and the independent variables – on the positive relationship linking empathic skills (independent variable) and the perceived impact of the Covid-19 outbreak on the economy (dependent variable) (direct effect: Z=2.37, p=0.02). Results highlighted a significant indirect effect of problem-oriented coping (Z=2.81, p=0.005), making up approximately 34% of the total effect of perceived social isolation on the perceived impact of the Covid-19 outbreak for health.

**Table 3:** Correlation analyses.

		*Outcomes*		*Global distress (DASS-21)*			*Loneliness (ILS)*			*Empathy (IRI)*		*Coping (COPE-NVI-25)*				
		*H*	*E*	*D*	*A*	*S*	*EL*	*SL*	*GL*	*EC*	*PT*	*PA*	*SS*	*PO*	*TO*	*AS*
*Outcomes*	*H*	–	0,502***	0,072*	0,154***	0,118***	-0,060*	-0,007	-0,88**	0,180***	0,059*	0,043	0,116***	0,0124***	0,099**	-0,29
	*E*		–	0,091***	0,068*	0,38	-0,015	0,009	-0,019	0,125***	0,079**	0,08**	0,096***	0,125***	0,093***	0,017
*Global distress (DASS-21)*	*D*			–	0,606***	0,738***	0,324***	-0,181***	0,352***	-0,025	-0,055	-0,110***	0,001	-0,125***	-0,010	0,261***
	*A*				–	0,656***	0,232***	-0,132***	0,227***	0,49	-0,005	0,066*	0,069*	-0,035	0,071*	0,219***
	*S*					–	0,291***	-0,124***	0,274***	0,024	-0,047	-0,085**	0,095***	-0,044	-0,008	0,180***
*Loneliness (ILS)*	*EL*						–	0,258***	0,837***	-0,27	-0,20	0,001	0,038	-0,061*	-0,063*	0,196***
	*SL*							–	-0,154***	0,241***	0,246***	0,272***	0,367***	0,293***	0,036	-0,122***
	*GL*								–	-0,096***	-0,065*	-0,021	-0,068*	-0,099**	-0,057	0,252***
*Empathy (IRI)*	*EC*									–	0,495***	0,318***	0,333***	0,346***	0,177***	-0,127***
	*PT*										–	0,424***	0,325***	0,402***	0,064*	-0,105***
*Coping (COPE-NVI-25)*	*PA*											–	0,440***	0,650***	0,178***	0,071*
	*SS*												–	0,571***	0,184***	0,049
	*PO*													–	0,177***	-0,097***
	*TO*														–	0,153***
	*AS*															–

The table reports correlation coefficients (Pearson's r) and statistical significance (*p<0.05; **p<0.0; ***p<0.005). *Variable acronyms: H, Perceived impact of COVID-19 on Health; E, Perceived impact of COVID-19 on Economy; D, Depression; A, Anxiety; S, Stress; EL, Emotional loneliness; SL, Social loneliness; GL, General loneliness; EC, Empathic concern; PT, Perspective taking; PA, Positive attitude; SS, Social support; PO, Problem orientation; TO, Transcendence orientation; AS, Avoidance strategies*.

## Discussion

The Covid-19 pandemic seems at present to be unstoppable, effecting countries all over the world. Although Italy is facing this extremely stressful situation with all the available weapons and tools, severe concern has arisen regarding the Italian national health system's capacity to take the brunt of any subsequent psychosocial and economic implications. To this purpose, recent data highlighted that a significant proportion of the Italian general population may have moderate-to-severe psychological distress during the early phases of the Covid-19 emergency (18).

In line with this evidence, baseline findings of the PsyCOVID study suggest that Covid-19 will represent a psychosocial catastrophe. On the one side, healthcare workers face the emergency not only at the physical level, as they are continuously exposed to the contagion and engaged in patient assistance and care, but they have to cope with a huge psychosocial burden. This requires healthcare professionals to put into play enormous resources to adapt themselves to the new dystopic situation, managing the increasing distress while trying to bring out the most effective coping strategy. On the other side, quarantine and other social distancing measures imposed by Italian authorities to the majority of the population can exacerbate feelings of loneliness and lack of connectedness in socially fragile individuals, as well as enhance the risk of negative mental health outcomes (8).

As with the SARS outbreak (19, 20), persistent psychological symptoms will affect healthcare personnel and outbreak survivors, families of affected patients, quarantined fragile individuals, and socially disadvantaged sub-populations (i.e., subjects affected by chronic disease, elderly population with mild cognitive impairments, aged people without close relatives). However, literature reports only a few studies investigating psychological variables related to the Covid-19 spread. Wang and co-workers (21) provided evidence of a moderate to severe psychological impact of the outbreak in more than half of Chinese respondents, with 16.5% of interviewed individuals having moderate to severe depressive symptoms, 28.8% moderate to severe anxiety symptoms, and 8.1% moderate to severe stress levels. Li et al. (22) reported self-control as a resilience factor potentially mitigating the perceived severity of the Covid-19 outbreak and mental health problems. Moccia and colleagues reported that the mental health burden due to the Covid-19 outbreak may be predicted by temperament and adult attachment style (8).

Here we provided a first look at the psychosocial effects that the Covid-19 outbreak is having in Italy, in the very first days after the Italian Government Decree #iorestoacasa of March 9, 2020. Our results about the perceived impact of the Covid-19 outbreak suggest that while the economy emergency is viewed as equally serious in all Italian regions and in both healthcare and non-healthcare workers, the health emergency is tightly linked to the professional status and the geographical spread of the Covid-19 outbreak. As expected, healthcare workers who have to deal with suffering and deaths day by day judged the health emergency as more serious than people not involved in Covid-19 patients assistance and care. At the same time, individuals living in Northern Italy—who are dramatically facing illness and suffering of close relatives and friends—felt the health emergency as more urgent than what individuals living in Central and Southern regions did.

Notably, we provided evidence that the severity of perception of the Covid-19 emergency reflected the individuals' psychosocial vulnerability. First, increased perceived social support (i.e., a low degree of loneliness) was significantly correlated to the increased perception of the Covid-19 impact on health. This suggests that the greater an individual's support network, is the worse (i.e., more serious) his/her judgment about the Covid-19 consequences for health will be. In other terms, having more people in our own social network increases the probability to have examples of positive or probable cases in mind [feeding the so-called representativeness heuristic (23)] and, thus, to consider the current emergency as more serious. This is particularly true for healthcare professionals who are continuously and physically in touch with patients and colleagues. However, such a relationship is mediated by perceived distress, contributing to nearly half of the total effect of loneliness on the perception of the Covid-19 impact on health.

*Model 2* highlighted that better empathic skills (i.e., how much better I can understand others' emotions and point of view) are related to a more serious perception of the Covid-19 impact on the economy. Such a result indicates that a profound understanding of what the restrictive measures mean for Italian entrepreneurs and organizations generates a more serious judgment of the impact of Covid-19 on the Italian economy. Interestingly, we observed that a third of the effect is mediated by problem-oriented coping strategies. This indicates that a way of facing problems based on active strategies, planning, and focused efforts towards problem resolution makes people more aware of the imminent crisis and gets them prepared to face the economic disaster (e.g. industrial conversion of activities to produce goods currently in high demand). Interestingly, a previous report on the psychosocial impact of global infection outbreaks, including the SARS and the West Nile Virus, showed that the perceived threat of these diseases were related with different coping strategies, also including empathic responding (24). In particular, the authors underlined that empathic responding is associated to an individual engagement in recommended health precautions and in the avoidance of behaviors entailing detrimental social and economical consequences. In this light, more empathic individuals may also display a higher adherence to the imposed measures, in order to prevent the contagion spread.

Crucially, psychosocial variables here investigated represent modifiable factors. The scientific literature provides a large range of intervention strategies and programs for each single domain (25–30). Of course, the day-by-day accurate reporting of the status of the epidemic and experts' opinion guidance on prevention and infection control play important roles in stabilizing people and overcoming the epidemic-related crisis. Actively mobilizing the population to participate in epidemic prevention and control can help to alleviate social anxiety and the feeling of helplessness and strengthen the sense of membership to a large community despite the physical distance and isolation due to restrictive measures. However, real-time updates of information on outbreak effects without more hopeful news to counteract this could be detrimental in the long time. This is the reason why, in such a catastrophic context, there is an urgent need to develop evidence-driven and multi-faceted intervention strategies to reduce adverse psychological impacts and psychosocial distress during, and especially after, the Covid-19 outbreak. Comparably, consensus guidelines to orient physicians, psychologists, and other mental care professionals toward an effective unified approach are urgent. The present work provides important suggestions that may help in defining new intervention programs. Our data, indeed, might help the government and health authorities to evaluate how and where to allocate resources in the future, including personnel, services, care facilities, and interventions, to manage the situation in the coming months and years.

Some criticisms in this report have to be underlined. First, we collected data *via* a self-administered survey, and thus possible issues might be related to recall bias and the intrinsic limitations of self-report measures. Secondly, the convenience sampling might have affected the generalizability of the present findings, as the sample cannot be considered actually representative of the Italian population. Another limitation is related to the cross-sectional nature of our report, which prevents the observation of changes of participants' perceptions of the Covid-19 impact over time. However, we are going to overcome such an issue by reporting longitudinal data after the planned follow-up assessment, once all the socially restrictive measures in Italy will definitively end. Moreover, we have to underline that, despite having confirmed our initial predictions by showing that the perceived impact of Covid-19 on health and the economy is affected by different psychosocial predictors, including distress and loneliness as well as empathic and coping abilities, there was the possibility that the opposite is also true. Further studies can address such a specific issue. Finally, future analyses will also benefit from taking into account the socio-economic status of participants, a variable that may be crucial to better understand individual differences in perceptions and psychosocial profiles.

In conclusion, only time will tell us whether Italian quarantine measures have prevented a historical disaster. However, the costs of the outbreak are not limited to medical aspects, as the virus has led to significant social, psychological, and economic effects globally. Our data teaches us the need to invest in preparedness to prevent, rapidly identify, and contain mid- and long-term consequences of global health emergency outbreaks such as Covid-19. Although reacting with travel bans and quarantines costs effort and economic resources and impacts on the well-being of millions of individuals cordoned off in a zone of contagion, it is reasonably necessary to contain further disasters. The psychological weight of thousands of suspected and confirmed Covid-19 cases and of huge numbers of deaths is difficult to bear without a known successful scenario. People are suffering from the weight of having a limited access to social or psychological support, as well as from not seeing a future constructive outlook.

In this view, big data analyses should analyze public health risks in the future in order to adjust health care strategies that could be implemented for any future crisis. We all need to move in this direction in order to understand and control the disease now and its effects later. Memories of the numbers of affected and diseased people will probably wane but psychosocial consequences will last. This modern war has just begun.

## Data Availability Statement

The raw data supporting the conclusions of this article will be made available by the authors, without undue reservation.

## Ethics Statement

The studies involving human participants were reviewed and approved by the University of Pavia and IUSS Pavia Ethics Committee. The patients/participants provided their written informed consent to participate in this study.

## Author Contributions

CCe, GS, CG, CCr: conception and design of the work. CCe, GS, CG, AD, CCr: acquisition, analysis of data. CCe, AD, SC, TV, CCr: interpretation of data. CCe, CCr: drafting the work. CCe, GS, CG, AD, SC, TV, CCr: revising and providing the final approval of the work.

## Conflict of Interest

The authors declare that the research was conducted in the absence of any commercial or financial relationships that could be construed as a potential conflict of interest.

## References

[1] Zhu N Zhang D Wang W Li X Yang MS Song J A Novel Coronavirus from Patients with Pneumonia in China, 2019. N Engl J Med (2020) 382(8):727–33. 10.1056/NEJMoa2001017 PMC7092803 31978945

[2] Din MAU Boppana LKT An update on the 2019-nCoV outbreak. American Journal of Infection Control (2020). 10.1016/j.ajic.2020.01.023 PMC7102631 32171622

[3] Albarello F Pianura E Di Stefano F Cristofaro M Petrone A Marchioni L 2019-novel Coronavirus severe adult respiratory distress syndrome in two cases in Italy: An uncommon radiological presentation. Int J Infect Dis (2020) 93:192–7. 10.1016/j.ijid.2020.02.043 PMC7110436 32112966

[4] Giovanetti M Benvenuto D Angeletti S Ciccozzi M The first two cases of 2019-nCoV in Italy: Where they come from? J Med Virol (2020) 92(5):518–21. 10.1002/jmv.25699 PMC7166327 32022275

[5] Spina S Marrazzo F Migliari M Stucchi R Sforza A Fumagalli R The response of Milan's Emergency Medical System to the COVID-19 outbreak in Italy. Lancet (2020) 395(10227):e49–50. 10.1016/S0140-6736(20)30493-1 PMC7124430 32119824

[6] Remuzzi A Remuzzi G COVID-19 and Italy: what next? Lancet (2020) 395:1225–28. 10.1016/S0140-6736(20)30627-9 PMC7102589 32178769

[7] Sim K Chua HC The psychological impact of SARS: a matter of heart and mind. CMAJ (2004) 170(5):811–2. 10.1503/cmaj.1032003 PMC343855 14993176

[8] Sani G Janiri D Di Nicola M Janiri L Ferretti S Chieffo D Mental health during and after the COVID-19 emergency in Italy. Psychiatry Clin Neurosci (2020) 74:372–3. 10.1111/pcn.13004 32248608

[9] Brooks SK Dunn R Amlot R Greenberg N Rubin GJ Social and occupational factors associated with psychological distress and disorder among disaster responders: a systematic review. BMC Psychol (2016) 4:18. 10.1186/s40359-016-0120-9 27114240 PMC4845476

[10] Brooks SK Dunn R Amlot R Rubin GJ Greenberg N A Systematic Thematic Review of Social and Occupational Factors Associated With Psychological Outcomes in Healthcare Employees During an Infectious Disease Outbreak. J Occup Environ Med (2018) 60(3):248–57. 10.1097/JOM.0000000000001235 29252922

[11] Welch SJ Twenty years of patient satisfaction research applied to the emergency department: a qualitative review. Am J Med Qual (2010) 25(1):64–72. 10.1177/1062860609352536 19966114

[12] Wang H Kline JA Jackson BE Laureano-Phillips J Robinson RD Cowden CD Association between emergency physician self-reported empathy and patient satisfaction. PloS One (2018) 13(9):e0204113. 10.1371/journal.pone.0204113 30212564 PMC6136813

[13] Khalid I Khalid TJ Qabajah MR Barnard AG Qushmaq IA Healthcare Workers Emotions, Perceived Stressors and Coping Strategies During a MERS-CoV Outbreak. Clin Med Res (2016) 14(1):7–14. 10.3121/cmr.2016.1303 26847480 PMC4851451

[14] Bottesi G Ghisi M Altoe G Conforti E Melli G Sica C The Italian version of the Depression Anxiety Stress Scales-21: Factor structure and psychometric properties on community and clinical samples. Compr Psychiatry (2015) 60:170–81. 10.1016/j.comppsych.2015.04.005 25933937

[15] Davis M Measuring individual differences in empathy: Evidence for a multidimensional approach. HJJop Psychol s (1983) 44(1):113. 10.1037/0022-3514.44.1.113

[16] Zammuner VL Italians' social and emotional loneliness: The results of five studies. JIJoSS (2008) 3(2):108–20. 10.5281/zenodo.1056172

[17] Foà C Tonarelli A Caricati L Fruggeri L COPE-NVI-25: validazione italiana della versione ridotta della Coping Orientation to the Problems Experienced (COPE-NVI). JPdS (2015) 2:123–40.

[18] Moccia L Janiri D Pepe M Dattoli L Molinaro M De Martin V Affective temperament, attachment style, and the psychological impact of the COVID-19 outbreak: an early report on the Italian general population. Brain Behav Immun (2020). 10.1016/j.bbi.2020.04.048 PMC7169930 32325098

[19] Maunder RG Was SARS a mental health catastrophe? [Editorial]. General Hospital Psychiatry (2009) 31(4):316–7. 10.1016/j.genhosppsych.2009.04.004 PMC7133640 19555790

[20] Mak IWC Chu CM Pan PC Yiu MGC Chan VL Long-term psychiatric morbidities among SARS survivors. JGhp (2009) 31(4):318–26. 10.1016/j.genhosppsych.2009.03.001 PMC7112501 19555791

[21] Wang C Pan R Wan X Tan Y Xu L Ho CS Immediate Psychological Responses and Associated Factors during the Initial Stage of the 2019 Coronavirus Disease (COVID-19) Epidemic among the General Population in China. Int J Environ Res Public Health (2020) 17(5):1729. 10.3390/ijerph17051729 PMC7084952 32155789

[22] Li J-B Yang A Dou K Cheung RY Self-control moderates the association between perceived severity of the coronavirus disease 2019 (COVID-19) and mental health problems among the Chinese public. PsyArXiv. (2020). 10.31234/osf.io/2xadq PMC7370094 32635495

[23] Tversky A Kahneman D Judgment under Uncertainty: Heuristics and Biases. Science (1974) 185(4157):1124–31. 10.1126/science.185.4157.1124 17835457

[24] Puterman E Delongis A Lee-Baggley D Greenglass E Coping and health behaviors in times of health crises: Lessons from SARS and West Nile. Global Public Health (2009) 4:69–81. 10.1080/17441690802063304 19153931

[25] Gardiner C Geldenhuys G Gott M Interventions to reduce social isolation and loneliness among older people: an integrative review. Health Soc Care Community (2018) 26(2):147–57. 10.1111/hsc.12367 27413007

[26] Falkenberg I Buchkremer G Bartels M Wild B Implementation of a manual-based training of humor abilities in patients with depression: a pilot study. Psychiatry Res (2011) 186(2-3):454–7. 10.1016/j.psychres.2010.10.009 21071099

[27] Masi CM Chen HY Hawkley LC Cacioppo JT. A meta-analysis of interventions to reduce loneliness. Pers Soc Psychol Rev (2011) 15(3):219–66. 10.1177/1088868310377394 PMC3865701 20716644

[28] Regehr C Glancy D Pitts A LeBlanc VR Interventions to reduce the consequences of stress in physicians: a review and meta-analysis. J Nerv Ment Dis (2014) 202(5):353–9. 10.1097/NMD.0000000000000130 24727721

[29] Sharma M Rush SE Mindfulness-based stress reduction as a stress management intervention for healthy individuals: a systematic review. J Evid Based Complementary Altern Med (2014) 19(4):271–86. 10.1177/2156587214543143 25053754

[30] Weisz E Zaki J Empathy building interventions: A review of existing work and suggestions for future directions. Jtohocs (2017) 205–17. 10.1093/oxfordhb/9780190464684.013.16

